# Seasonal Xylem Sap Acidification Is Governed by Tree Phenology, Temperature and Elevation of Growing Site

**DOI:** 10.3390/plants11152058

**Published:** 2022-08-06

**Authors:** Manuel Pramsohler, Edith Lichtenberger, Gilbert Neuner

**Affiliations:** 1 Laimburg Research Centre, Laimburg 6, Pfatten/Vadena, 39040 Auer/Ora, Italy; 2 Unit of Functional Plant Biology, Department of Botany, University of Innsbruck, Sternwartestraße 15, 6020 Innsbruck, Austria

**Keywords:** acidification, alkalinisation, bud burst, freezing, *Malus domestica*, pH, *Picea abies*, *Pinus cembra*

## Abstract

pH of xylem sap (pHx) was determined in three trees (*Malus domestica* (apple tree)*,* *Picea abies* and *Pinus cembra*) in response to seasonal changes. Conifer trees from lowland (600 m) were compared to trees growing at the alpine timberline (1950 m a.s.l.). Xylem sap was extracted with a Scholander pressure bomb and pHx was measured with a pH microsensor. In all species, pHx changed markedly with season. In spring, pHx was acidic; during winter, the pHx was more alkaline. In apple trees, the pHx did not show a significant correlation with temperature but was rather affected by developmental stage. During flushing in spring, xylem sap acidification took place concomitant to the developmental stage “tight cluster”, when foliar development enables a significant transpiration and a consequent movement of water in the xylem. The xylem sap of the two studied conifers showed a significantly larger seasonal alkalinisation (+2.1) than found in apple trees (+1.2) and was significantly more pronounced at the timberline. Xylem sap acidification took place before bud break. pHx had a significant negative correlation with soil temperatures and corresponded to already reported pHx of angiosperms. Overall, pHx appears to be a sensitive stress marker and indicator of activity status in tree xylem.

## 1. Introduction

The pH of the xylem sap (pHx) of plants ranges from acidic values of 4.5 to about 7.4 [1], which is in contrast to the milieu of the symplast, which has an alkaline pH ranging from 7.2 to 7.5 [2]. Only a few studies report on seasonal changes of pHx. In deciduous temperate trees, xylem sap usually becomes more alkaline in winter and then acidifies during spring [3,4,5,6,7,8,9]. In contrast, it can also be the other way round, as in *Juglans regia* pHx acidifies during winter and becomes more alkaline in spring [10].

Various mechanisms are reported to be responsible for the regulation of the pHx. All factors that affect proton pump activity [8,11,12] and xylem sap composition, especially the concentration of cations/anions and organic compounds, might be involved [13,14,15]. In addition, the pHx influences the concentration of dissolved CO_2_ in the xylem sap and therefore regulates the amount of CO_2_ that can be transported through the xylem in trees [1,9,16]. Seasonal pHx changes in deciduous species may be linked to the onset of the ascent of xylem sap, which is coupled to transpiration in the foliated stage. Unfortunately, studies reporting on seasonal changes of pHx in deciduous trees lack information on phenology and short-term variations during flushing in spring. For evergreen trees and gymnosperms, where the end of winter dormancy and the ascent of xylem sap are not linked to the formation of new leaves, only very few reports about seasonal changes of pHx are currently available. In conifers growing at the alpine timberline a marked pHx alkalinisation during winter is reported [17].

Seasonal pHx changes are likely the consequence of steadied water and nutrient transport in the xylem that, in turn, is under environmental control and affected by winter stress. Under laboratory and field conditions, several abiotic and biotic stresses have been shown to cause an alkalinisation of the xylem or apoplastic sap, including drought [18,19,20], salt stress [21,22], flooding [23,24], chilling temperatures [25] and fungal infection [25,26]. Additionally, it has been shown that environmental factors that influence transpiration rates, such as increased solar irradiation, vapour pressure deficit (VPD) and increased temperatures can influence the pHx [16,27,28]. Particularly in response to drought, alkalinisation of the xylem sap induces accumulation of the phytohormone abscisic acid (ABA) in the leaf apoplast triggering stomatal closure [18,20,29]. During winter, soil frosts can induce drought stress, which becomes even more severe at high elevations, where winter desiccation is a widespread phenomenon observed in woody plants [30]. Little is known about the effects of subalpine winter environmental conditions on the alkalinisation of the pHx.

The aims of the present study were (1) to determine seasonal changes of pHx in a deciduous angiosperm (apple tree, *Malus domestica*) and in two evergreen gymnosperm tree species (*Picea abies*, *Pinus cembra*) and to correlate pHx values with seasonal temperature changes, (2) to compare seasonal changes of pHx in the same species under contrasting environmental conditions and growing sites (greenhouse versus field conditions in apple trees in order to advance tree phenology, low versus high elevation in evergreen gymnosperms) and (3) to assess the short-term dynamic of pHx in relation to developmental phenology during flushing of apple trees in spring.

## 2. Results

**Figure 1:**
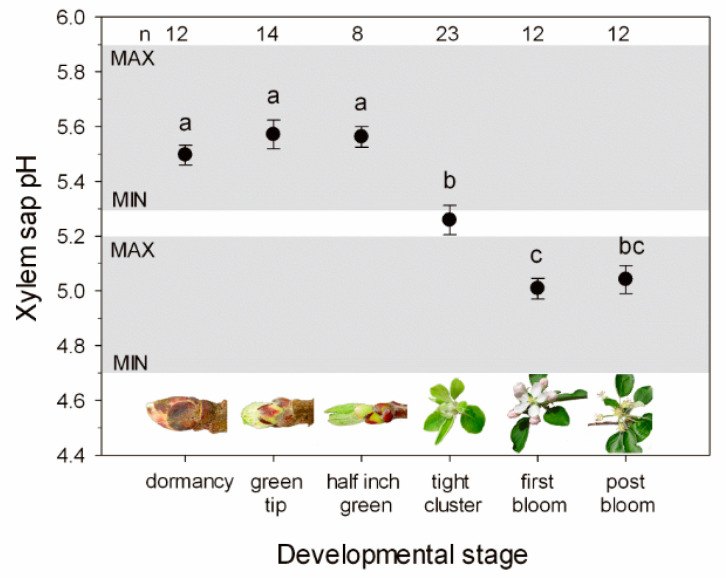
Seasonal change of pHx of twigs of *M. domestica* as related to the developmental stage of buds during flushing in spring. The grey boxes indicate the minimum and maximum for the respective season. Data (mean values ± SE, replicate numbers are given in the figure) are from December to May from two measurement years and were obtained on potted trees and orchard trees at field sites. Different letters indicate significant differences (tested by ANOVA and the Tamhane test at *p* < 0.05). The pictures illustrate the phenology or stage of bud development.

The pHx of apple trees was found to change significantly with season, with a mean value of pH 5.0 ± 0.03 in spring and pH 5.6 ± 0.05 in winter (Figure 1). The range between the minimum (pH 4.7) and maximum pH value (pH 5.9) recorded during the two measurement years was 1.2 pH units. In winter, the xylem sap was more alkaline. Acidification of the xylem sap occurred during regrowth in spring and was strongly linked to the developmental stage of buds during flushing. While in the early developmental stages, “green tip” and “half inch green”, the pH remained unchanged; in later stages, beginning with a “tight cluster”, the pH of the xylem sap decreased significantly (*p* < 0.05). In all later developmental stages, pHx values were more acidic and significantly different from the early developmental stages.

The onset of or release from soil frost had no effect on pHx in apple trees. The seasonally occurring temperature fluctuations (mean values of soil and air temperatures 7 days before sampling) did not have any immediate effect on pHx of apple trees (see Table 1).

**Figure 2:**
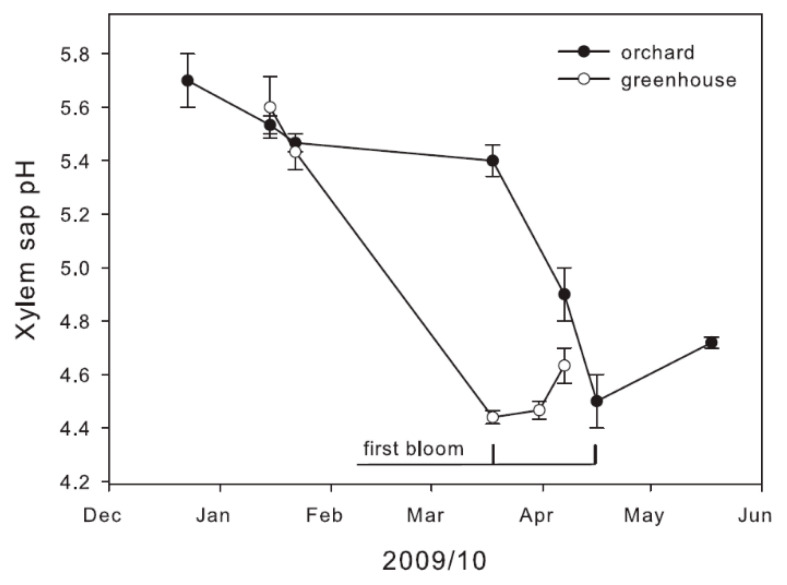
Seasonal change of pHx of twigs of potted *M. domestica* trees grown in an orchard (closed circles) or under greenhouse conditions (10/25 °C; open circles). Data are mean values ± SE (*n* = 4). Trees were transferred into the greenhouse on 15 January 2010.

Similar seasonal pHx changes were obtained for potted apple trees exposed to two contrasting temperature conditions (Figure 2). The range between mean values measured in spring (pH 4.4 ± 0.02) and mean values measured in winter (pH 5.7 ± 0.1) was 1.3 pH units. In potted trees, pHx values in spring were slightly more acidic than in trees from the field site. In the trees exposed to greenhouse conditions xylem sap acidification and flushing occurred earlier. In the greenhouse, apple trees were already at the developmental stage of “first bloom” in the middle of March. In the field, potted trees started to bloom nearly one month later.

**Figure 3:**
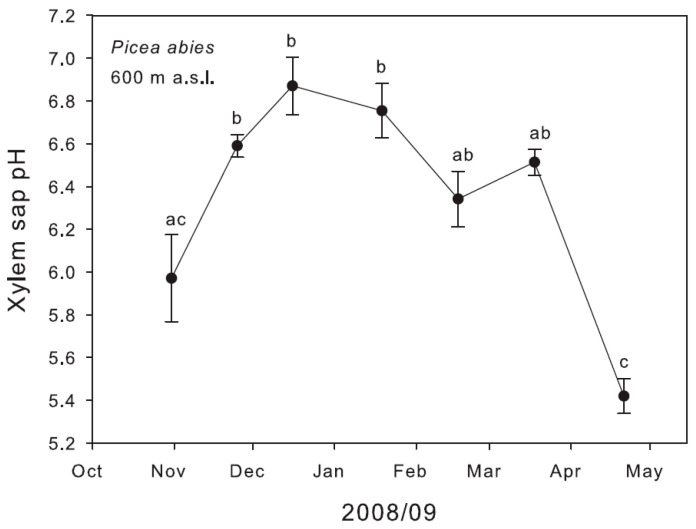
Seasonal change of pHx of twigs of *P. abies* sampled in the Botanical Garden in Innsbruck at 600 m a.s.l. Different letters indicate significant differences between mean values (±SE; *n* = 5) tested by ANOVA and the Bonferroni test at *p* < 0.05.

In the investigated evergreen gymnosperms, a significant seasonal change of pHx was also found. In *P. abies* at 600 m a.s.l. alkalinisation in winter was more pronounced than in apple with mean values of pH 5.4 ± 0.08 in spring and pH 6.9 ± 0.13 in winter (Figure 3). The range between the minimum (pH 5.2) and maximum pH value (pH 7.3) measured was 2.1 pH units. In *P. cembra* at 600 m a.s.l., mean values of pH 6.1 ± 0.3 in spring and pH 6.8 ± 0.07 in winter were found (data not shown).

**Figure 4:**
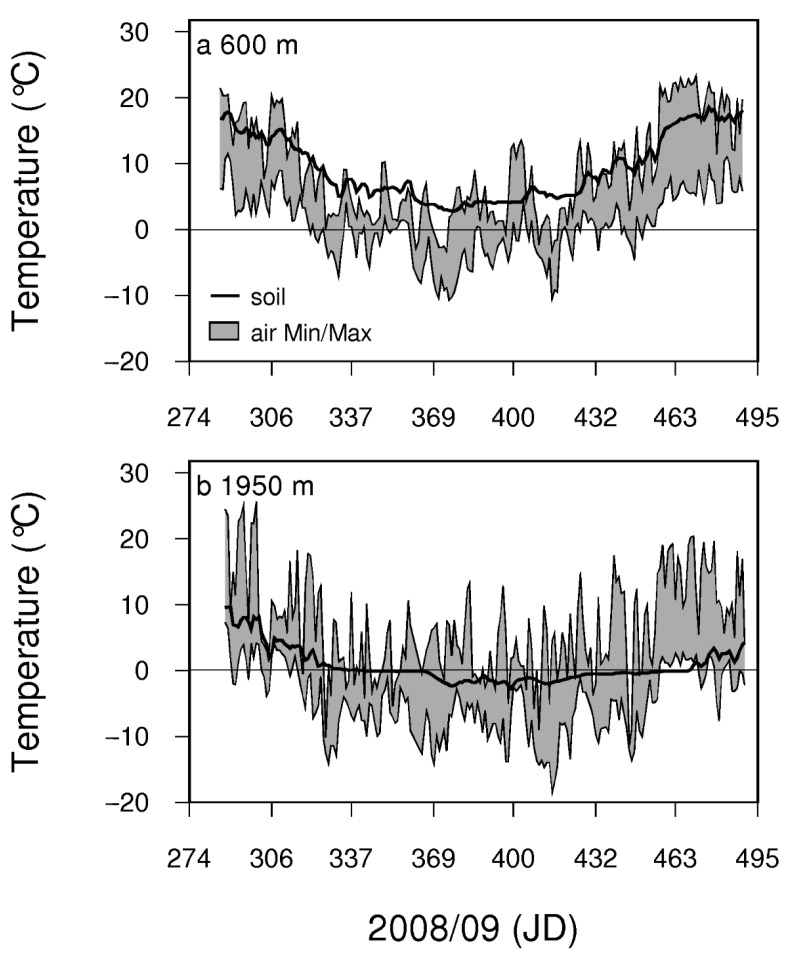
Temperature conditions in the Botanical Garden at 600 m a.s.l. (**a**) and at the field site at the timberline at 1950 m a.s.l. on Mt. Patscherkofel (**b**). The solid line shows the daily mean soil temperature at 5 cm depth, the grey area marks the daily minimum and maximum air temperatures measured at a height of 2 m.

**Figure 5:**
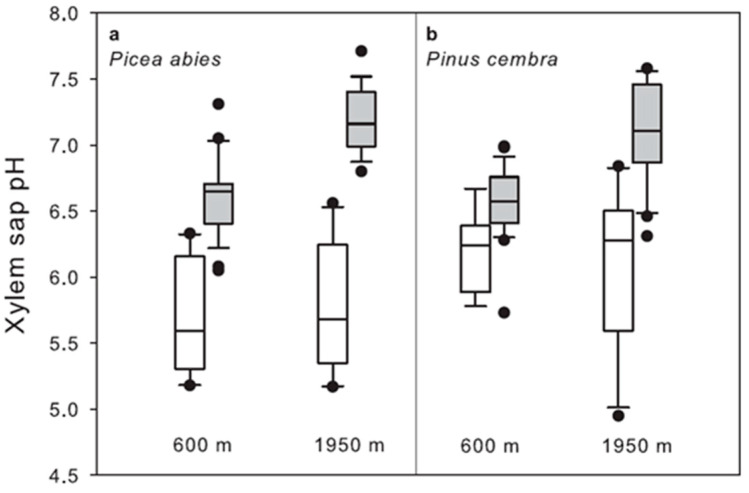
Seasonal change of pHx of (**a**) *P. abies* and (**b**) *P. cembra* twigs sampled at 600 m a.s.l. in comparison to samples from the alpine timberline at 1950 m. White boxes show values obtained during the growing period (*n* = 9), grey boxes show winter values (*n* = 25). The boxplots present the median and the 10th, 25th, 75th and 90th percentiles; outliers are shown as dots. For both species at both growing sites winter values were significantly different (Bonferroni at *p* < 0.05) from values obtained during the growing period. Winter alkalinisation was significantly higher (*t*-test at *p* < 0.001) at 1950 m for both species.

Soil and air temperatures at the two contrasting field sites clearly differed (Figure 4). Particularly at the high elevation site (1950 m a.s.l.), there was a prolonged soil frost period during winter. Winter xylem sap alkalinisation of *P. abies* and *P. cembra* from lowland (600 m a.s.l.) and from the timberline ecotone sites (1950 m a.s.l.) are compared in Figure 5. In winter, xylem sap was more alkaline at the growing site at 1950 m independent of species (*p* < 0.001). In both evergreen gymnosperms, a significant alkalinisation was observed during winter at both elevations, with a stronger alkalinisation at the high elevation growing site (*p* < 0.001). pHx values in the gymnosperms showed a negative correlation with the seasonally occurring air and soil temperatures (*P. abies* at 600 m and soil temperature r = −0.901, *p* < 0.05 see Table 1). In contrast to apple trees, the xylem sap of the evergreen gymnosperms acidified before bud break in spring.

## 3. Discussion

In the three investigated species, the pHx showed a pronounced seasonal variation. While in spring and autumn, pHx was acidic, a significant alkalinisation took place during winter. The measured pHx values were in the range of other published values for pHx [1,19]. The alkalinisation measured over winter corroborates earlier observations for a number of temperate deciduous angiosperm trees (*Acer pseudoplatanus* pH 5.4–6.9 [3]; *Actinidia chinensis* pH 5.3–6.2 [4]; *Betula pendula* pH 5.7–7.5 [5]; *Populus × canadensis “robusta”* pH 5.4–7.5 [6]; *Fagus sylvatica* pH 4.8–6.7 [31]; *Robinia pseudoacacia* pH 5.2–6.0 [8]).

In spring and autumn pHx of the gymnosperms *P. cembra* and *P. abies* was acidic. Acidification was also reported for other gymnosperms (pH 5.3 in *Abies koreana* [32]; pH 5.6 in *Pinus taeda* [33]). The seasonal dynamic of pHx in gymnosperms has recently been studied [17]; as for deciduous angiosperms, we found a significant winter alkalinisation in evergreen gymnosperms. The seasonal pHx amplitude in the two studied evergreen gymnosperms was higher than in apple trees, but still in the range of the reported maximum seasonal amplitude of 2.3 pH units as reported for *Fagus sylvatica* [7,31]. Our results allow us to compare between the studied species but not between the functional groups of trees. Further studies with a higher number of conifer and deciduous tree species are needed to compare between functional groups of trees at different environments.

In apple trees, xylem sap acidification in spring was linked to the bud developmental stage “tight cluster” and there was no significant correlation between xylem sap acidification and the seasonally occurring soil or air temperatures. A close relationship between developmental stages during flushing and pHx values was found in trees grown under field conditions and individuals exposed to greenhouse conditions. At the onset of bud break, when the release from winter dormancy first becomes visible, no significant changes of pHx values were found. In the developmental stage of “tight cluster” when foliation had proceeded to such an extent that a significant transpiration and consequent ascent of water and nutrients in the xylem were possible, pHx began to decrease. Concomitant measurements of transpiration and xylem conductivity reveal that in the developmental stage of “tight cluster” the developing leaves are already transpiring; however, xylem hydraulic conductance is not yet fully restored [34,35].

For apple trees, significant seasonal variations in the quantitative mineral and amino acid composition of the xylem sap are reported [36,37]. Moreover, for *P. abies*, the mineral composition of the xylem sap and its seasonal variation are reported [38,39]. In the investigated evergreen gymnosperms xylem sap acidification in spring occurred before bud break and therefore before the new needles of the current season emerged. We assume that in evergreen species the absence of water movement in the xylem in winter is directly related to winter stress and low environmental temperatures; therefore, a correlation between pHx values and temperatures can be found. In deciduous tree species, transpiration and water movement in the xylem can start only after foliation in spring and therefore pHx values might not be directly related to the environmental temperatures in spring. Tree phenology is linked to occurring environmental temperatures and therefore climate change can lead to phenological shifts [40]. These shifts in tree phenology will directly influence the timing of the seasonal variations in pHx for apple trees. In the two studied conifers at the alpine timberline, a correlation between pHx and the seasonal occurring soil temperatures was found. Increasing temperatures due to climate change will therefore also affect the seasonal changes of pHx in timberline conifers.

The underlying mechanisms for the seasonal variation of pHx are not fully understood. All factors affecting proton pumping activity might be involved [11]. Fromard et al. [8] showed that in *Robinia pseudoacacia* the plasma membrane H^+^-ATPase of the vessel-associated cells is responsible for the control of pHx, and its activity changes with season. In winter the activity of the H^+^-ATPase was low and therefore xylem sap was more alkaline. Seasonal changes in the amount of the H^+^-ATPase in cambial and expanding xylem cells are reported for twigs of two *Populus* species [12]. Furthermore, the xylem sap composition has been shown to influence the pHx due to the buffering capacity of distinct components [14,15,41,42]. In the xylem sap of beech roots, a significant correlation between low pH values and high concentrations of Ca^2+^, Mg^2+^ and malate was found [13]. The author concluded that the increase in malic acid, which forms complexes with cations, is responsible for the springtime acidification of the xylem sap in beech roots.

In the case of the winter acidification of the xylem sap of *Juglans regia* [10], the pHx values were not directly related to the activity of the H^+^-ATPase of vessel-associated cells, but depended on the seasonal variation of the sugar content in the xylem sap. Proton-coupled active sugar transport mechanisms were shown to be responsible for this [10,43,44]. There is a strong relationship between pHx and the concentration of dissolved CO_2_ in the xylem sap. Therefore, pHx regulates the amount of CO_2_ transported through the xylem and, furthermore, the exchange of CO_2_ between the different stem tissues [1,45,46]. Knowledge about the species-specific xylem sap pH might be useful for the calculation of CO_2_ budgets of individual trees.

The comparison of pHx values of the same species from contrasting elevations clearly revealed that at 1950 m a.s.l. winter alkalinisation was more pronounced than at 600 m in both investigated gymnosperms. The amount of mean winter alkalinisation in *P. abies* was 1.5 pH units at 600 m compared to 2.0 pH units at 1950 m. Similarly, in *P. cembra* we measured a mean winter alkalinisation of 0.6 pH units at 600 m compared to 1.5 pH units at 1950 m. In spring, pHx values were unaffected by elevation in the two gymnosperms. In both species at both growing sites, a significant correlation between pHx values and soil temperatures was found. At the timberline during winter the soil was frozen for a prolonged period causing winter drought stress that increased in severity with duration [30]. Timberline conifers are growing at the upper distribution boundaries of the respective tree species. Information on pHx and xylem sap composition may help to understand the survival mechanisms of the respective tree species at the timberline [17]. Under laboratory and field conditions, drought has been repeatedly identified as a factor in xylem sap alkalinisation, due to a reduced proton pumping activity, leading to the accumulation of abscisic acid (ABA) in the leaf apoplast and inducing stomatal closure [11,18,27,28,47]. The alkalinisation of the xylem sap in response to drought stress is not a universal mechanism in higher plant species [32]. However, the response to the apoplastic alkalinisation—elevated ABA concentration in the apoplast and induction of stomatal closure—is thought to be a universal mechanism in plants [32]. The winter alkalinisation of the xylem sap in evergreen gymnosperms might be part of a physiological stress response in evergreen trees, which keeps the stomata securely closed during winter. Nevertheless, irrespective of its physiological function, pHx can be nicely used to assess the current activation state of the xylem tissue in trees.

## 4. Materials and Methods

### 4.1. Study Site and Plant Material

Seasonal changes of the pHx were studied in a deciduous (apple tree, *Malus domestica* Borkh. cv. “Golden Delicious” growing on “M9” rootstock) and two evergreen tree species (*Picea abies* L. Karst. and *Pinus cembra* L.). Samples from apple trees were either taken from trees growing in an apple orchard in Tarsch, Italy (46°36′ N, 10°53′ E, 860 m a.s.l., 20 trees were used for sampling) or from 20 potted trees cultivated in the Botanical Garden, University of Innsbruck, Austria (47°16′ N, 11°23′ E, 600 m a.s.l.). To break dormancy ahead of field sites and to advance tree phenology, four potted apple trees were transferred into a greenhouse (10/25 °C night/day) on 15 January 2010. pHx values of these trees growing in the greenhouse were then compared to potted trees kept under field conditions. Apple trees were from 5 to 6 years old, and 2-year-old and 3-year-old shoots were used for xylem sap extraction. Samples from the evergreen gymnosperms were taken from two different field sites with contrasting elevations: at 600 m a.s.l. from trees growing in the Botanical Garden of the University of Innsbruck, and from trees growing close to the timberline ecotone on Mt. Patscherkofel (1950 m a.s.l.; 47°12′ N, 11°27′ E). Xylem sap samples from *P. abies* and *P. cembra* (twigs with a diameter of 0.7–0.9 cm, detached from adult trees) were taken from the end of October 2008 to the end of May 2009. Apple trees were sampled over two years from December 2009 to May 2011. Twig samples were always taken between 10.00 and 12.00 a.m.

### 4.2. Temperature Conditions

At the field sites, soil and air temperatures were recorded with sets of type T thermocouples. Soil temperatures were measured at a depth of 5, 10 and 20 cm, air temperatures were measured at a height of 2 m. Thermocouples were connected to a multiplexer (AM16/32B, Campbell Scientific, Logan, UT, USA) and temperatures were recorded at 7 min intervals with a CR10 data logger (Campbell Scientific, Logan, UT, USA). Temperatures of the potted apple trees in the greenhouse experiment and in the field were measured every 30 min with TidBit temperature data loggers (Onset, Pocasset, MA, USA).

### 4.3. Xylem Sap Collection

For xylem sap extraction, twigs with a diameter of 0.7–0.9 cm were used. Twig samples were detached from the trees and transported in plastic bags from the field sites to the laboratory. Samples were either prepared for immediate measurement or stored overnight in a cold room at +4 °C until measurements started. When twigs from the alpine timberline site were frozen at the time of sampling, they were stored overnight at −8 °C in commercial freezers until the beginning of measurements. Xylem sap was collected using a Scholander pressure bomb (Model 3115, Soil moisture Equipment Corp., Santa Barbara, CA, USA). Twigs were cut into pieces of a mean length of about 15 cm. The bark, including the phloem and the cambium layer, was peeled off to ensure that the expressed liquid came only from the xylem. The pieces of wood were then sealed into the lid of the pressure chamber. After closure of the pressure chamber, the pressure was gradually increased to a maximum pressure of between 0.2 and 2.5 MPa. Xylem sap leaking out at the cut surface of the wood was then collected with glass capillaries until the volume was sufficient for pH measurements. pHx was measured immediately after xylem sap collection. This sampling methodology may affect xylem sap composition [48]. To minimise this aspect, we applied only the minimum pressure necessary for xylem sap collection and worked with pH microsensors that allowed measurements with small quantities of xylem sap (see following paragraph).

### 4.4. pH Measurements

In apple twigs the pHx of the expressed xylem sap was measured with two different pH sensor types. With a needle-type pH microsensor (pH-1 micro, PreSens, Regensburg, Germany) measurements could be conducted inside glass capillaries (inner capillary diameter: 1.15 mm), which were used to suck up xylem sap. The pH-1 micro was connected to a PC for digital registration of the pH values. Because of the small dimension of the sensor (sensor tip < 150 µm), measurements were possible even within sap volumes < 0.02 mL. The second sensor type employed to measure apple xylem sap pH was a PHR-146 micro electrode (Lazar Research Laboratories, Los Angeles, CA, USA). For measurements with this sensor type the extracted xylem sap was also collected within glass capillaries, but then transferred to microtiter plates and measured in the microtiter plate. One droplet of xylem sap (0.05 mL) was sufficient for pH measurement. The pH sensor was connected to a digital pH meter (Jenco Model 60, Jenco Instruments, San Diego, CA, USA) and pH values could be read from the display. Calibration of the pH sensors was performed with certified buffer solutions (Merck, Darmstadt, Germany) according to the user instructions of the respective pH sensor. Additionally, all recordings were cross checked with non bleeding pH-indicator strips (Merck, Darmstadt, Germany). Measurements of the xylem sap of gymnosperms were taken using a micro combination pH electrode (Amani-1000, Innovative Instruments, Tampa, FL, USA). This sensor has a tip diameter of 1 mm and was also injected into the glass capillaries (inner diameter 1.6 mm) containing the collected xylem sap. The sensor was connected to a data logger (CR10X, Campbell Scientific, Logan, UT, USA) for digital registration of the values. Between 4 and 5 twig samples were used for the pHx measurements.

### 4.5. Tree Phenology

Classification of floral bud phenology in apple trees was conducted according to the descriptions given in the BBCH scale [49]. In the text the following terminology is used: “dormancy” (BBCH 00), “green tip” (BBCH 53), “half inch green” (BBCH 54), “tight cluster” where leaves are unfolding (BBCH 56), “first bloom” (BBCH 60) and “post bloom” (BBCH 69). In the two studied conifer species the timing of bud break in spring was observed.

### 4.6. Statistical Data Analysis

pHx values are given as mean ± standard error of the mean (SE). After values passed the Kolmogorov–Smirnoff and the Levene tests, significant differences between mean values were tested using one-way ANOVA and the Bonferroni post hoc test (*p* < 0.05). If homogeneity of variances was not established, significant differences between mean values were tested with one-way ANOVA and the Tamhane test. pHx values of gymnosperms from the two contrasting elevations were compared using Student’s *t*-test. Correlations between mean pHx values and the seasonally occurring air and soil temperatures were calculated using Pearson’s correlation analysis. All analyses are carried out using PASW Statistics 18 (formerly SPSS, IBM Corporation, New York, NY, USA). Replicate numbers are given in the figures or in the figure legends.

## Figures and Tables

**Table 1:** Effect of air and soil temperatures (mean values from 7 days before sampling) on pHx (mean values, *n* = 5). The Pearson correlation coefficient (r) is given for calculations obtained with data from eight different sampling dates.

Species	Air Temperature		Soil Temperature	
	R	*p*	R	*p*
*Malus domestica* ^1^	−0.345	0.402	−0.375	0.407
*Picea abies* ^2^	−0.887	0.003	−0.901	0.002
*Pinus cembra* ^2^	−0.669	0.069	−0.756	0.03
*Picea abies* ^3^	−0.768	0.075	−0.924	0.008
*Pinus cembra* ^3^	−0.660	0.153	−0.826	0.043

^1^ measured on twig samples in an apple orchard from October 2010 till May 2011; ^2^ measured on twigs sampled at 600 m a.s.l. from October 2008 till April 2009; ^3^ measured on twigs sampled at 1950 m a.s.l. from October 2008 till April 2009.
